# Biophysical Characterization of Essential Phosphorylation at the Flexible C-Terminal Region of C-Raf with 14-3-3ζ Protein

**DOI:** 10.1371/journal.pone.0135976

**Published:** 2015-08-21

**Authors:** Anirban Ghosh, Bhisma Narayan Ratha, Nilanjan Gayen, Kamal H. Mroue, Rajiv K. Kar, Atin K. Mandal, Anirban Bhunia

**Affiliations:** 1 Department of Biophysics, Bose Institute, P-1/12 CIT Scheme VII (M), Kolkata, 700 054, India; 2 Department of Molecular Medicine, Bose Institute, P-1/12 CIT Scheme VII (M), Kolkata, 700 054, India; 3 Biophysics and Department of Chemistry, University of Michigan, Ann Arbor, Michigan, 48109–1055, United States of America; Griffith University, AUSTRALIA

## Abstract

Phosphorylation at the C-terminal flexible region of the C-Raf protein plays an important role in regulating its biological activity. Auto-phosphorylation at serine 621 (S621) in this region maintains C-Raf stability and activity. This phosphorylation mediates the interaction between C-Raf and scaffold protein 14-3-3ζ to activate the downstream MEK kinase pathway. In this study, we have defined the interaction of C-terminal peptide sequence of C-Raf with 14-3-3ζ protein and determined the possible structural adaptation of this region. Biophysical elucidation of the interaction was carried out using phosphopeptide (residue number 615–630) in the presence of 14-3-3ζ protein. Using isothermal titration calorimetry (ITC), a high binding affinity with micro-molar range was found to exist between the peptide and 14-3-3ζ protein, whereas the non-phosphorylated peptide did not show any appreciable binding affinity. Further interaction details were investigated using several biophysical techniques such as circular dichroism (CD), fluorescence, and nuclear magnetic resonance (NMR) spectroscopy, in addition to molecular modeling. This study provides the molecular basis for C-Raf C-terminal-derived phosphopeptide interaction with 14-3-3ζ protein as well as structural insights responsible for phosphorylated S621-mediated 14-3-3ζ binding at an atomic resolution.

## Introduction

Raf protein belongs to the Raf-MEK-ERK protein kinase signaling pathway that controls various cellular functions, including cell proliferation, growth, differentiation and survival [1]. As the upstream activator of ERK signaling, Raf proteins thus play an integral role in MAPK pathway regulation and its biological effects; Raf kinase is thus tightly regulated for optimum downstream signaling. Dysregulation of Raf signaling causes aberrant ERK activation and develops several pathogenic conditions [2–6]. Among the three isoforms of Raf proteins, A-Raf, B-Raf and C-Raf found in vertebrates, C-Raf has been studied extensively for its regulation and pathway activation. C-Raf activation is governed by phosphorylation/dephosphorylation events, Ras binding, membrane recruitment, along with intra- and inter-molecular interactions with other proteins [7–12]. The complex regulation process of C-Raf involves the recruitment of dimeric protein 14-3-3ζ that binds to C-Raf in a phosphorylation-dependent manner. Residues S233 and S259 in the regulatory domain [13,14] and S621 in the C-terminus of C-Raf catalytic domain [15,16] are the primary target of 14-3-3ζ that keeps C-Raf in an inactive conformation inside the cytosol [17]. Upon mitogenic stimulation, dephosphorylation of S259 residue by protein phosphatase 2A (PP2A) leads to the release of 14-3-3ζ from pS233 and pS259 site [18], which results in translocation of C-Raf to the membrane and activates the downstream effector MEK kinase (MAP2K) [19–21].

The C-terminus of C-Raf catalytic domain plays an important role in regulating C-Raf activity. This domain possesses an auto-phosphorylation residue S621 [22–25], of which phosphorylation is essential for C-Raf stability [24], and has been shown as both inhibitory and activating [15,16,23,26,27]. Substitution of S621A is kinetically inactive, affects ATP binding without affecting MEK interaction [25], and reduces the capacity of hetero-dimerization [28]. 14-3-3ζ recruitment to this pS621 residue activates C-Raf [15,16], possibly stabilizing the kinase in its active conformation. The interaction between certain kinases with 14-3-3ζ is found to be involved in the regulation of their functioning [29–32]. Perturb interaction shows significance reduction in signaling mediated by C-Raf kinase [15,26]. Due to lack of structural details from the C-terminus region of C-Raf, the driving factors behind the strong binding of 14-3-3ζ to the C-terminus tail of C-Raf remain elusive.

**Fig 1:**
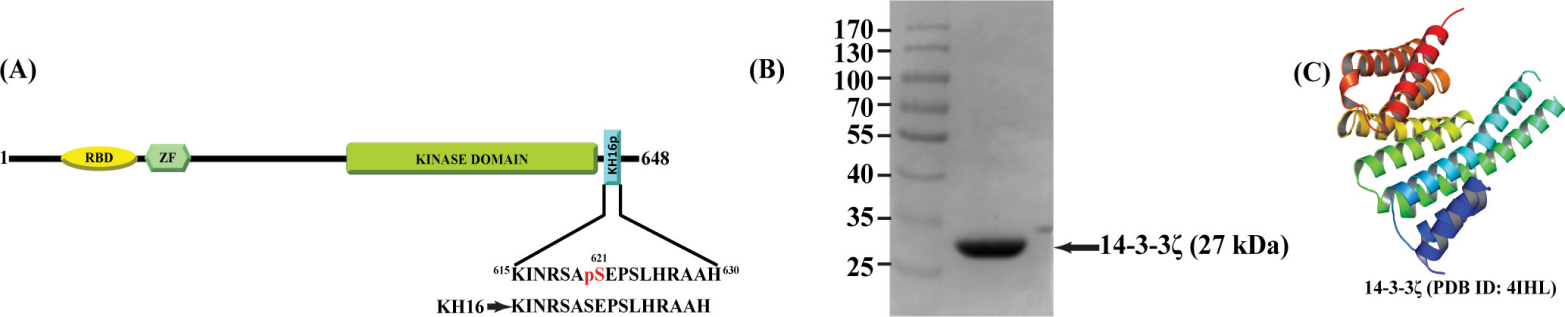
Sequence of KH16p with structure and purification of 14-3-3ζ protein. **(A)** Schematic presentation of C-Raf protein showing the sub-domains RBD (RAS-binding domain), ZF (Zinc Finger domain), Kinase domain, and KH16p (615–630 residues). pS621 is indicated in red color (denoted as KH16p), and the control peptide KH16 devoid of phosphorylarion at S621 is shown below. (**B)** Purification of 14-3-3ζ protein. Human 14-3-3ζ protein was cloned into pQE30 vector containing N-terminal His-tag. *E*. *Coli* M15 strain was transformed with this clone and 14-3-3ζ protein was expressed after induction with 0.5 mM IPTG for 3 hours. 14-3-3ζ protein was purified by Ni^2+^-NTA resin. 10 μg of purified 14-3-3ζ protein was loaded into SDS-PAGE and stained with Coomassie brilliant blue. (**C)** Crystal Structure of human 14-3-3ζ (PDB ID: 4IHL, Chain A) demonstrating α-helical structure.

The original aim of this study was to use NMR spectroscopy for determining the three-dimensional structure of C-Raf extreme C-terminus using pS621 peptide (610–648 residues) in the presence of 14-3-3ζ protein. However, our preliminary results exhibited strongly overlapping and unresolved peaks in the NMR spectra obtained from this domain. To overcome the potential difficulties and to obtain an in-depth insight into the biophysical basis of peptide-protein interaction, we have chosen the phosphopeptide stretch 615^th^-630^th^ (where S621 residue is phosphorylated, hereafter denoted as KH16p) (Fig 1A) that contains the signature sequence (RSXpS^621^XP) for 14-3-3ζ binding. Our results show a high binding affinity of KH16p (pS621) peptide to 14-3-3ζ protein identical to that of the pS259 peptide with 14-3-3ζ [17]. The phosphate residue of S621 is stabilized by the positively charged groove of the 14-3-3ζ protein, and the peptide adopts a random coil structure similar to that observed previously with an 8-residue peptide (618^th^-625^th^) [33]. We have used multiple biophysical methods such as circular dichroism (CD), fluorescence, NMR and isothermal titration calorimetry (ITC) to elucidate the interaction between protein and phosphopeptide motif at an atomic resolution, which are corroborated by molecular modeling studies. The comprehensive investigations presented in this study offer ample opportunity to acquire more in-depth insights into various important aspects of S621-mediated 14-3-3ζ binding, which potentially can enable us to shed more light on the kinase activation process.

## Materials and Methods

### Cloning

Human 14-3-3ζ full length sequence was PCR amplified using pcDNA3 14-3-3ζ as a template with forward primer (5´-CTCCCTCGGATCCATGGATAA-3´) and reverse primer (5´-TGCACTGCAGCGGGGTCTACTGTG-3´). The PCR amplified product was digested with BamH1 and Pst1 and cloned into pQE30 vector. This vector contains N-terminal hexa- histidine tag suitable for affinity purification. The cloned fragment was confirmed by automated DNA sequencer, Applied Biosystem 3130×I 96 capillary DNA Analyzer.

### Purification of 14-3-3ζ protein

*E*. *coli* M15 cells were transformed with this construct and grown at 37°C in LB medium containing ampicillin and kanamycin. The cells were grown till 0.6 (O.D._600nm_) and the expression of the protein was induced with 0.5 mM IPTG for 3 hours. The cells were harvested and stored at -70°C. The cells were subsequently resuspended in lysis buffer (10 mM NaH_2_PO_4_, 100 mM NaCl, pH7.0, 1 mM PMSF (Sigma, St. Louis, MO), 1X Halt Protease inhibitor cocktail (ThermoFisher Scientific, Waltham, MA) and lysed by passing through the French press at 20,000 psi. Cell lysate was centrifuged at 20,000×g for 15 minutes at 4°C. The supernatant was collected and again centrifuged at 100,000×g for 1 hour. Cell homogenate was applied onto TALON affinity resin (Clontech, Mountain View, CA) pre-equilibrated with lysis buffer. Resin was washed with five volumes of lysis buffer containing 20 mM imidazole. Bound protein was eluted with 150 mM imidazole containing lysis buffer. Imidazole was then removed by dialysis; the buffer was exchanged with 10 mM NaH_2_PO_4_, 100 mM NaCl, pH 6.1 and the protein was concentrated in sucrose bed. Commercially synthesized and HPLC-purified (> 95%) KH16p and KH16 peptides were purchased from GL Biochem (Shanghai, China) and used as received.

### Circular Dichroism

Secondary structures of KH16p peptide and 14-3-3ζ protein were studied with Jasco J-815 spectrophotometer. 10 mM Na_2_HPO_4_ buffer containing 100 mM NaCl (pH 6.1) was used throughout the study. Quartz cuvette with a path length of 0.2 cm was used to record CD spectra at a temperature of 298 K for 25 μM KH16p in the absence as well as in the presence of 14-3-3ζ protein (1 μM). Spectra were recorded over a range of 195–260 nm at 0.1 nm data interval with a scanning speed of 100 nm/min. Each CD spectrum represents an accumulation of four subsequent scans. To eliminate contributions of 14-3-3ζ protein in the CD spectrum, the same concentration of protein CD spectrum (without KH16p) was subtracted from the resultant CD spectra. The blank buffer data (in millidegrees) were subtracted from the collected spectral raw data and converted to molar ellipticity using Eq (1):

$$Molar\;ellipticity\;\left(\theta \right)=\;\frac{m_{0}M}{10}\;L\;\times \;C$$ (1)

Where *m*_0_ is millidegrees, *M* is the molecular weight (g mol^−1^), *L* is the path length of quartz cuvette used (cm), and *C* is the concentration (Molarity).

The CD spectral data were further analyzed using CDNN software (version 2.1) (http://bioinformatik.biochemtech.uni-halle.de/cdnn/) for deconvolution purposes, as described elsewhere [34,35].

Temperature-induced denaturation from 278 K to 368 K of 14-3-3ζ protein (5 μM) and in the presence of KH16p at 1:2 molar ratio was studied by applying same experimental parameters. The calculation for percentage helicity was followed from previously published work [36,37]. The melting temperatures (T_m_) of the protein were calculated using a three-parameter sigmoidal fitting.

### Fluorescence Experiments

Fluorescence experiments were performed using Hitachi F-7000 FL spectrometer with a 0.1 cm path length quartz cuvette at 298 K. 14-3-3ζ and KH16p stock solutions were prepared in 10 mM Na_2_HPO_4_ buffer containing 100 mM NaCl at pH 6.1. Since 14-3-3ζ contains two Trp residues in its sequence, the excitation wavelength was chosen at 295 nm to selectively excite Trp residues and emission in a range of 310–400 nm. An excitation and emission slit of 5 nm was used [38]. To observe KH16p and 14-3-3ζ binding interaction, 5 μM 14-3-3ζ protein was titrated with increasing concentration of KH16p at a molar ratio up to 1:5.

### Isothermal Titration Calorimetry

ITC technique was employed to study thermodynamics of KH16p and KH16 peptides interactions with 14-3-3ζ protein with the help of VP-ITC micro-calorimeter in high feedback mode. The peptides were dissolved in the same dialysis buffer of 14-3-3ζ protein (i.e., 10 mM Na_2_HPO_4_ buffer containing 100 mM NaCl, pH 6.1). Sample cell with 50 μM 14-3-3ζ protein was titrated against 1 mM peptide in a syringe with 300 *rpm* stirring speed with an initial delay of 60 sec. The cell temperature was kept at 298 K and 10 μcal/s of reference power as applied to maintain a flat baseline. A total of 17 injections of 1 mM KH16p/KH16 were performed, where the initial injection was of 0.4 μl and the final was 2.0 μl, while the remaining 15 injections were 2.5 μl each. Each injection was over a period of 0.8 sec with a spacing of 150 sec and filter time of 5 sec. An equivalent control experiment to subtract the heat of dilution of the peptides in buffer was performed (i.e., the same concentration of peptide was injected into the buffer solution keeping same experimental parameters). Results obtained were plotted using MicroCal Origin 7.0 software. In order to determine the thermodynamic parameters (i.e., association constant *K*_*A*_ and enthalpy of reaction Δ*H*), the single-site binding model was used. Other thermodynamic parameters such as the free energy of binding (Δ*G*) and entropy (Δ*S*) were calculated using Eqs (2) and (3), respectively:

$$\Delta G=-RTln\;K_{A}$$ (2)

ΔG=ΔH−TΔS (3)

### Nuclear Magnetic Resonance (NMR) Experiments

NMR experiments were carried out at either 288 K or 298 K on a Bruker AVANCE III 500 MHz NMR spectrometer, equipped with a 5 mm SMART probe. Experimental data were collected and processed with Topspin v3.1 software (Bruker, Switzerland). One-dimensional ^1^H NMR spectra, two-dimensional total correlation spectroscopy (TOCSY) and transferred nuclear overhauser effect spectroscopy (*tr*NOESY) spectra of 1 mM KH16p peptide in 10 mM Na_2_HPO_4_ buffer with 100 mM NaCl at pH 6.1 containing 10% D_2_O were recorded after titrating with 14-3-3ζ protein (3.3 μM final). The spectral widths were set to 12 ppm in both dimensions, and mixing times for NOESY and TOCSY experiments were kept at 150 ms and 80 ms, respectively. DSS (4,4-dimethyl-4-silapentane-1-sulfonic acid) was used as an internal standard for all NMR experiments. The sequential assignment from 2D TOCSY and NOESY spectra was performed using Sparky software (T. D. Goddard and D.G. Kneller, University of California, San Francisco).

One dimensional proton-decoupled ^31^P NMR spectra were recorded at 288 K at a peptide concentration of 400 μM in deionized water at pH 6.1 with various concentrations of 14-3-3ζ protein (0, 2, 6, 12, 20, 30, and 40 μM). Spectra were recorded with 512 scans and 16 dummy scans with a 10 sec recycle delay. A 20 Hz exponential line broadening function was applied to each spectrum prior to Fourier transformation.

Saturation transfer difference (STD) NMR experiments were carried out at 288 K using standard STD pulse sequences, with WATERGATE 3-9-19 water suppression method [39,40]. Briefly, 1 mM of KH16p/KH16 peptide in 10 mM Na_2_HPO_4_ buffer (pH 6.1) with 100 mM NaCl was lyophilized and resuspended in an equal volume of D_2_O, and 2 μM 14-3-3ζ proteins was added to it. A train of 40 selective Gaussian-shaped pulses (49 ms each) with an interval period of 1 ms was used to saturate protein resonance for a total of 2 sec saturation time. During the STD NMR experiment, on- and off-resonance frequencies were set at -1 ppm and 40 ppm, respectively. Subtraction of off-resonance spectrum from the on-resonance spectra to yield signals appearing due to saturation transfer from protein to ligand was achieved using phase cycling [41]. The one-dimensional STD spectra were acquired with 1024 scans with 16 dummy scans using a sweep width of 12 ppm. An exponential line broadening function of 3 Hz was applied to the spectral data before Fourier transformation. Similar STD experiments were performed on the peptide KH16p or KH16 in the absence of 14-3-3ζ protein as a control.

### Molecular Modeling Studies

Molecular modeling was carried out to predict the structure of peptide KH16p and its interaction with the 14-3-3ζ protein. Peptide structure was prepared using the build option in Maestro and addition of phosphate group was done at S7 (S621 of C-Raf) position of KH16p. The peptide was solvated in orthorhombic water box containing TIP3P water models [42]. The prepared peptide structure was processed for conformational sampling simulated annealing using OPLS_2005 force-field [43]. The time frame used for simulated annealing was 5 ns, after which the production run for 15 ns at a constant temperature of 300 K was used for the conformational sampling of explicit solvent medium. Full details of the process are found in a previously published report [44]. Final structures of peptides were analysed with respect to energy component to select conformation for further studies. Lowest energy structure was adopted for docking process.

The peptide and 14-3-3ζ protein model (PDB accession code 4IHL; chain A) [33] were subjected to protein preparation wizard [45,46]. This process helps to avoid potential problems associated with overlapping atoms, bond order, and/or missing atoms. Water molecules present in the X-ray crystal structure were removed during the preparation. Patchdock [47] server was used for obtaining the complex of KH16p peptide and 14-3-3ζ protein, with all the default value settings. Validation of the docked complexes was done in conjunction to the STD NMR results [40]. The obtained complex was then solvated in TIP3P water models in orthorhombic boundary conditions. The adopted protocol and parameters used for minimization and equilibration steps are similar to previous reports [44]. A production run of 15 ns was set with NTP ensemble using a cut-off value of 10 Å. Trajectory frames were saved at an interval of 5 ps for subsequent analysis.

## Results

### Peptide Design

Previous studies have shown that S621 phosphorylation in the C-terminus of C-Raf kinase domain is essential for its activity [48]. S621 is an auto-phosphorylation site and the phosphorylation of this residue occurs in a *cis* manner [24]. The significance of this phosphorylation for maintaining C-Raf activity is still not fully understood owing to the flexibility of this region, which hinders the structure determination of intact kinase domain. Attempts have been made by co-crystallizing phosphorylated peptide (618–625) with 14-3-3ζ that interacts with pS621 residue, and obtained a random coil structure [33]. It is noteworthy to mention that the short peptide (8 residues) is less likely to adopt a secondary structure. In addition, on many occasions, the protein-peptide complex crystal structure may vary in solution [49]. In light of this, we have chosen a stretch of 16 amino acids (615–630, KH16p) of C-Raf around phosphorylated S621 to determine its solution structure in the presence of 14-3-3ζ protein, as shown in Fig 1A. We have purified the homogeneous human 14-3-3ζ protein in bacteria (Fig 1B). The structural stability of 14-3-3ζ protein in the presence of the KH16p peptide was studied in order to acquire more insights into the thermodynamics of the interaction between KH16p and 14-3-3ζ protein.

### Determination of secondary structure of 14-3-3ζ protein in the presence of KH16p peptide and *vice versa*

**Fig 2:**
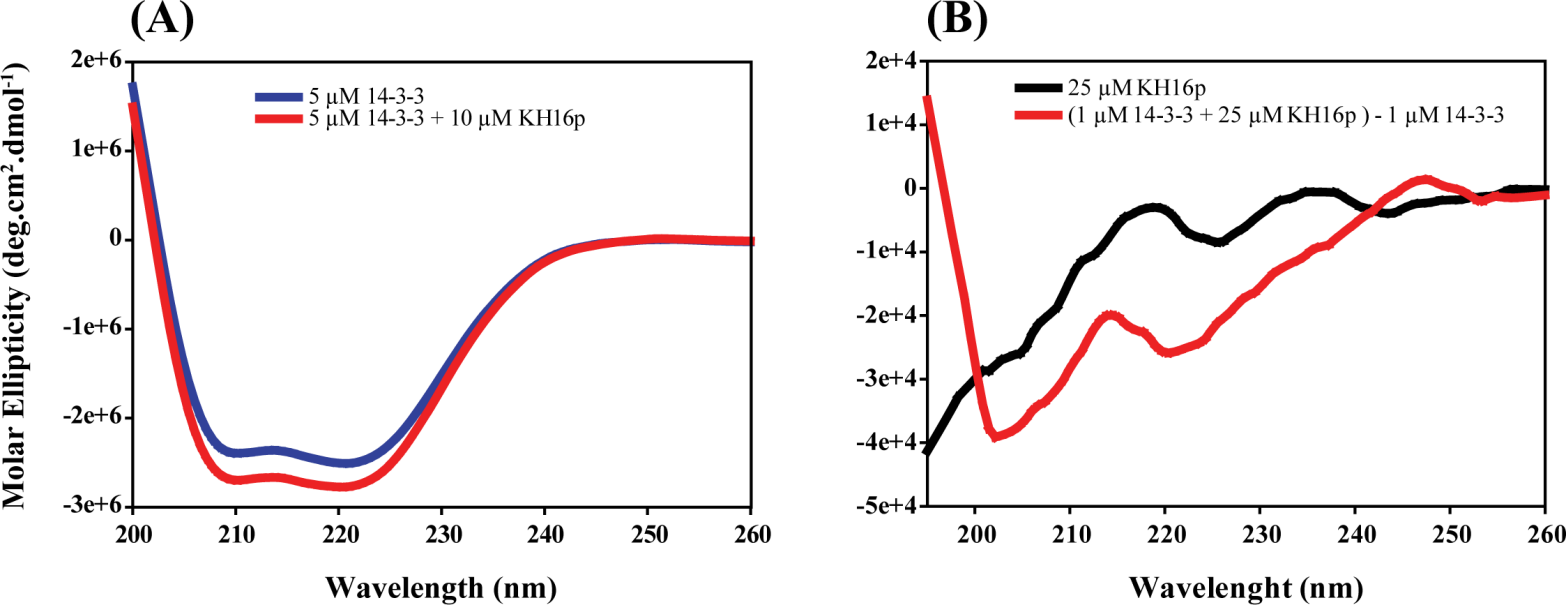
Relative changes in secondary structure of 14-3-3ζ protein upon interaction with KH16p peptide and *vice versa* by CD spectroscopy. **(A)** Far-UV CD spectra of 14-3-3ζ protein in free and KH16p-peptide bound forms. (**B)** Far-UV CD spectra of KH16p peptide in absence and presence of 14-3-3ζ protein. Protein and peptides were in 10 mM Na_2_HPO_4_ buffer with 100 mM NaCl at pH 6.1.

Circular dichroism is a useful tool for rapid characterization of the secondary structure of a biomolecule. The X-ray crystal structure of 14-3-3ζ protein features nine alpha helices and one 3_10_ helix (Fig 1C). The far CD studies of 14-3-3ζ protein reveal the same helical secondary structure of the 14-3-3ζ protein. It can be observed in Fig 2A that the far CD signature of 14-3-3ζ protein consists of two sharp minima at 222 nm and 208 nm, which are due to (n > π*) and (π > π*) transitions of the amide chromospheres. In the presence of the KH16p peptide at a 1:2 molar ratio, the intensities of the two negative maxima were further increased. This gain in intensity can be attributed to a considerable interaction between KH16p peptide and 14-3-3ζ protein. However, no significant changes in secondary structure occur, which confirms the retention of protein α-helicity. This spectral pattern reveals stabilization of 14-3-3ζ protein secondary structure due to its interaction with KH16p peptide which is further probed by deconvolution of CD spectral data. We have found that while the relative contribution of helix % increases, the random coil % decreases whereas the parallel, anti-parallel and beta turn % remain almost unchanged (**Fig A in S1 File**).

On the other hand, CD spectra of KH16p show random coil conformation, as evident from the negative maxima at ~195 nm due to n > π* transition (Fig 2B). Interestingly, in the presence of 1 μM 14-3-3ζ protein, there was a significant change in the spectral signature. The negative maxima have shifted to ~205 nm along with another smaller negative shoulder at ~220 nm. This can be attributed to a gain in partial helicity of the KH16p conformation in presence of 14-3-3ζ protein, along with subpopulation of other minor conformations. Based on the CD deconvolution results, we have found that the % of helicity has greatly increased (24%) while that of anti-parallel turn has decreased (22%) keeping other conformation % approximately the same (**Fig A in S1 File**). Taken together, our CD data suggest no major conformational changes in 14-3-3ζ protein; rather, it retains its helical structure, whereas the KH16p peptide may adopt some ordered confirmation in comparison to the free peptide.

### Thermal denaturation of 14-3-3ζ protein and effect of KH16p on denaturation

Thermal denaturation study of the protein in the presence of KH16p peptide was conducted in order to gain insight into the structural details of the interaction. The melting temperature (T_m_) value for the 14-3-3ζ protein alone was found to be 331 K (58.12 ± 0.47°C), while T_m_ increased to 332.5 K (59.70 ± 0.60°C) in the presence of KH16p. This data indicate that the protein gains thermal stability in the presence of KH16p as its T_m_ increases by ~1.6°C (**Fig B in S1 File**). This complements our previous CD spectral data, which confirms that 14-3-3ζ protein is stabilized by binding to KH16p peptide.

### Thermodynamics of KH16p interaction with 14-3-3ζ protein from ITC experiments

**Fig 3:**
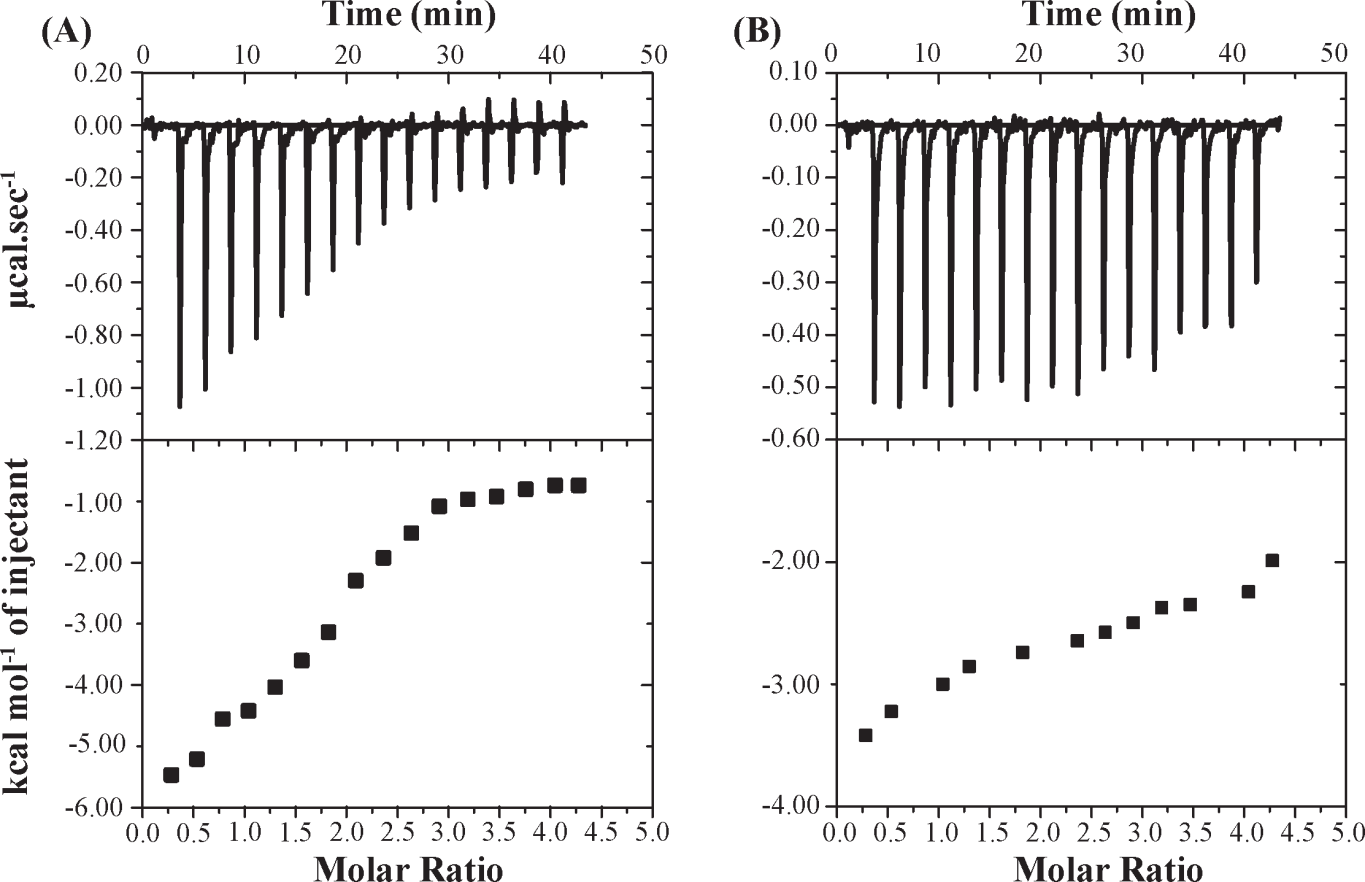
Isothermal titration calorimetric (ITC) profile of KH16p (A) and KH16 (B) interaction with 14-3-3ζ protein. Upper panels represent the exothermic heat of reaction plotted against time (minutes), and the lower panels represent the enthalpy change per mole of injected peptide plotted against the molar ratio of peptide to protein. KH16p peptide and 14-3-3ζ protein were in 10 mM Na_2_HPO_4_ with 100 mM NaCl (pH 6.1) buffer at 298 K.

Isothermal titration calorimetry (ITC) is an established tool to obtain thermodynamic insight of biomolecular interactions at a particular temperature. Thermodynamic parameters for the interaction of KH16p peptide with 14-3-3ζ protein were obtained by fitting the ITC experimental data to one set of single-binding site model (Fig 3A), and are summarized in Table 1. The downward direction in the ITC thermogram suggests exothermic heat burst during the binding event. It is evident from the magnitude of *K*_D_ that the relevant interaction is in the micro-molar (μM) range. The signs of Δ*H* (*-*6557 ± 295.6 cal.mol^-1^) and Δ*S* (-0.209 cal.mol^-1^.deg^-1^) also provide the thermodynamic signature of the process. Hence, the binding interaction of KH16p and 14-3-3ζ protein is entropically unfavorable (Δ*S* < 0) and enthalpy driven (Δ*H* < 0). The interaction of KH16p with 14-3-3ζ is termed spontaneous in nature due to the negative value of the Gibbs free energy of binding (Δ*G* = - 6551.775 ± 295.6 cal.mol^-1^). Such enthalpy-driven processes can be attributed either to electrostatic interactions or to hydrogen-bond formation underlying the binding phenomenon [50]. We have also performed a control ITC experiment with the non-phosphorylated peptide (KH16) and found that it binds to the 14-3-3ζ protein with a much lesser affinity (Fig 3B). In addition, the ITC thermogram shows a negligible heat change with respect to time, which precludes the calculation of other relevant thermodynamic parameters. Overall, the ITC experiment successfully probed the binding of KH16p with 14-3-3ζ protein, whereas KH16 peptide did not show any appreciable binding affinity with the 14-3-3ζ protein. This in turn highly indicates that the strong electrostatic interaction between phosphate group (pS621) of KH16p peptide and positively charge amino-acid residues (such as Arg or Lys) of 14-3-3ζ may play an important role in mediating the binding phenomenon.

**Table 1:** ITC-derived thermodynamic parameters for the interaction between KH16p and 14-3-3ζ protein.

Peptide	*N*	*K*_A_(M^-1^)	∆*H*(cal mol^-1^)	∆*S*(cal mol^-1^ deg^-1^)	*T*∆*S*(cal mol^-1^)	∆*G*(cal mol^-1^)	*K*_D_
**KH16p**	1.94 ± 0.06	5.77 × 10^4^± 8.87 × 10^3^	*-*6557±295.6	-0.209	-5.225	-6551.775	17.33 μM
**KH16**	1.89 ± 1.45 × 10^4^	85.0 ± 8.04 × 10^4^	-4.132 × 10^5^ ± 3.549 × 10^9^	-1.38 × 10^3^	-411.24 × 10^3^	-1.96 × 10^3^	117 mM

### ^31^P NMR spectroscopy

**Fig 4:**
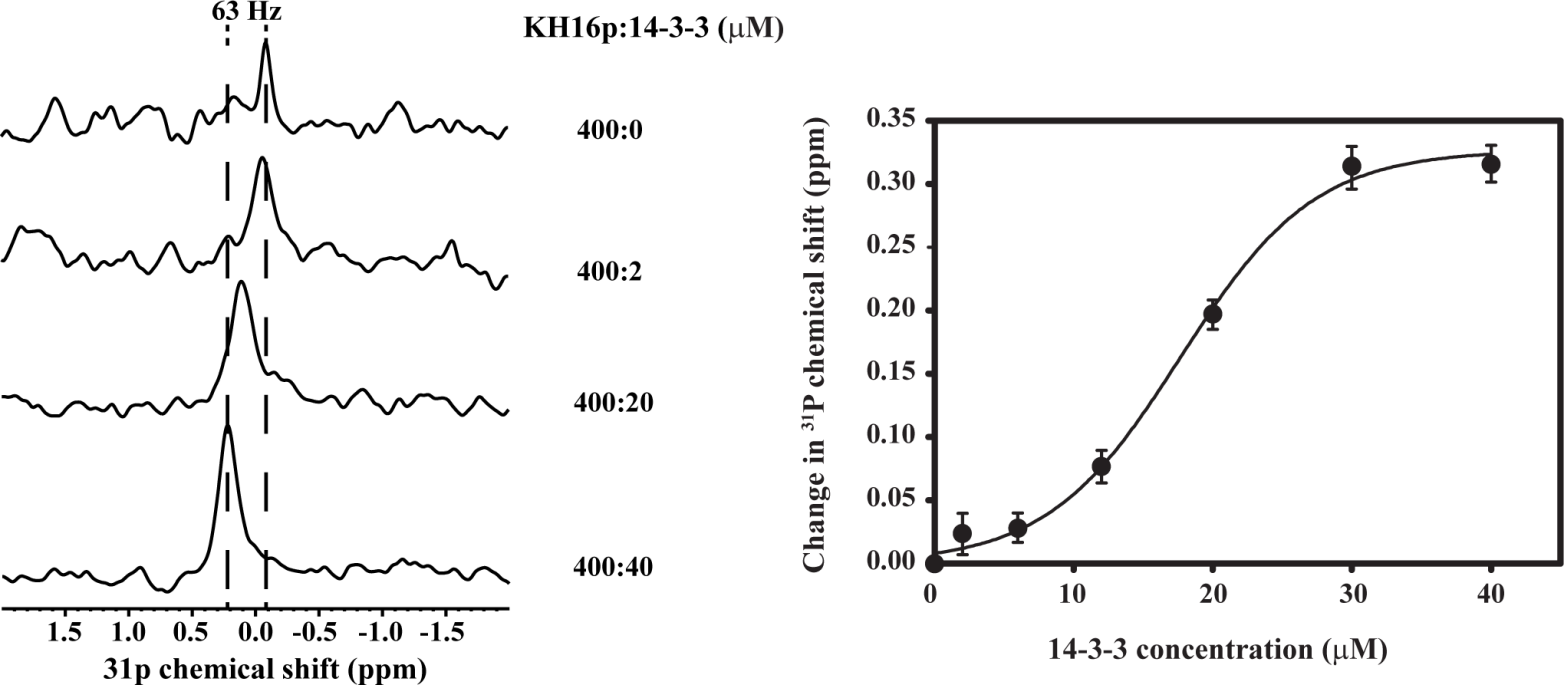
^31^P NMR spectra displaying KH16p interaction with 14-3-3ζ protein. The left panel demonstrates changes observed in ^31^P NMR spectra of KH16p upon addition of protein in a concentration-dependent manner, and the right panel shows the sigmoidal fitted plot for the change in ^31^P NMR chemical shift as a function of protein concentration.

^31^P NMR can be used as a sensitive probe to detect electrostatic interactions present in biomolecular events [51]. Due to the presence of phosphorylated serine group in KH16p, we have used ^31^P NMR experiments to investigate the nature of its electrostatic interaction with the14-3-3ζ protein. The ^31^P NMR spectra of KH16p showed a single peak at about -0.1 ppm upfield. The subsequent addition of 14-3-3ζ protein in a concentration-dependent manner causes a remarkable change in the chemical shift of ^31^P in the NMR spectra, as can be observed in Fig 4. The downfield shift in ^31^P NMR resonances becomes prominent with increasing concentration of 14-3-3ζ protein. This effect can be attributed to the electrostatic interaction between phosphate groups and Lys/Arg side chains present in the protein. The shift in ^31^P NMR chemical shift is plotted against the concentration of protein added, which yields a sigmoidal curve (Fig 4). The estimated *K*_D_ value (17.7 ± 0.6 μM) from this curve matches well with the ITC-derived value (*K*_D_ = 17.3 μM).

### ^1^H NMR spectroscopy

**Fig 5:**
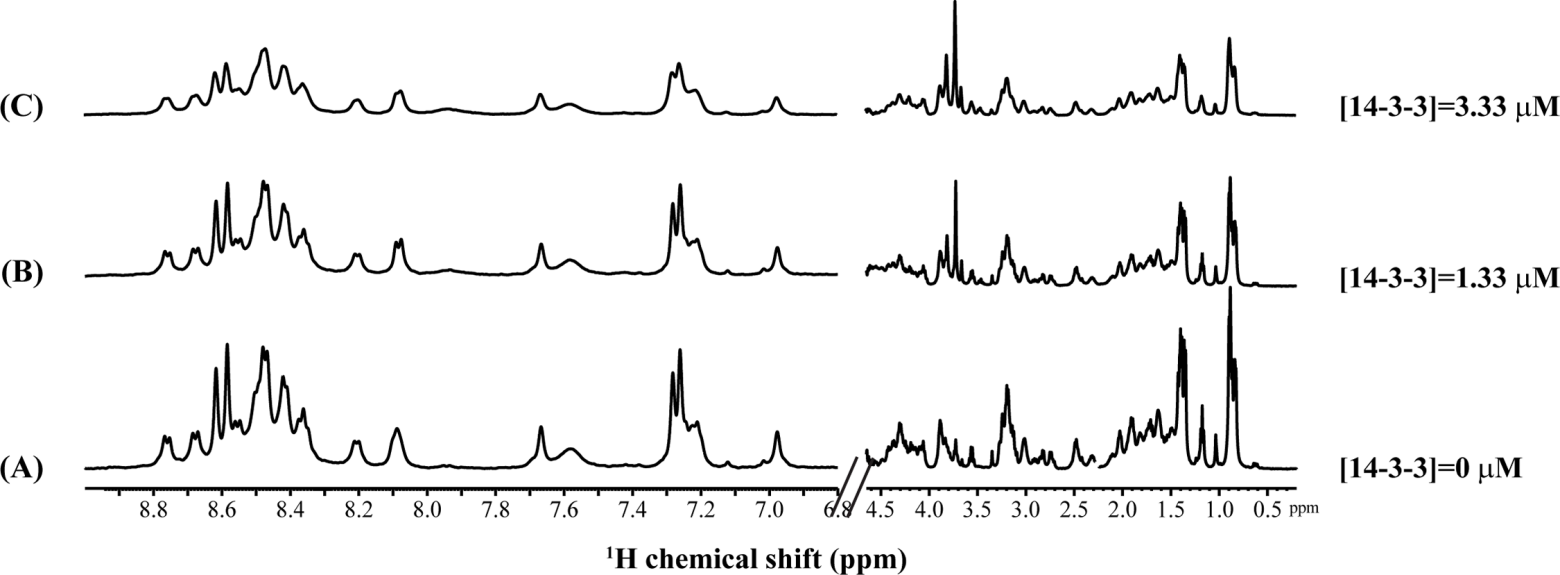
Interaction of KH16p with 14-3-3ζ using ^1^H NMR spectroscopy. ^1^H NMR spectra of the KH16p peptide with an increasing concentration of 14-3-3ζ demonstrating the linewidth broadening in the amide, aromatic, and aliphatic regions. The ^1^H NMR experiments were carried out in aqueous solution on a Bruker AVANCE III 500MHz spectrometer at 288 K.

Fig 5 shows the one-dimensional ^1^H NMR spectra of KH16p in the presence of increasing concentrations of 14-3-3ζ protein up to 3.33 μM. The addition of low concentration of 14-3-3ζ protein (~1 to 3 μM) to KH16p (~1 mM) showed concentration-dependent line-width broadening of amide, aromatic and aliphatic proton resonances without any chemical shift perturbation. The amount of broadening was estimated to be 5–7 Hz, indicating a fast chemical exchange between free and bound forms on the NMR time scale [52]. Notably, this condition is ideal for *transferred* Nuclear Overhauser effect spectroscopy (*tr*NOESY) experiments to elucidate the three-dimensional structure of the protein-bound ligand/peptide [53]. *tr*NOESY is a well-established NMR method for determining the three-dimensional protein-bound conformations of ligand molecules that undergo fast to intermediate exchange between their free and bound states, with dissociation constants (*K*_D_) in the micromolar to millimolar range [51,54]. Two-dimensional TOCSY and NOESY spectrum were used for the complete sequence-specific resonance assignment for KH16p bound to 14-3-3ζ. The NOE cross peak between any amino-acid alpha hydrogen (CαH) and its preceding-residue amide proton (NH) has been traced out and shown in **Fig C in S1 File**. Surprisingly, aromatic ring protons of histidine residue also did not show any notable NOE cross peaks between the side chain of the hydrophobic amino acids and their aromatic ring protons (data not shown). Due to the presence of sequential NOEs [αN (i, i+1)] only, it precludes the formation of any regular secondary structure such as alpha helix or beta sheet. The trans-conformation of the E8-P9 peptide bond was identified because of the presence of NOE contacts between E8 CαH/P9 CδH cross peak (data not shown) [55]. Determination of the structure of KH16p was not possible due to the presence of significant overlap and a small number of NOE cross peaks in the NOESY spectra.

### STD NMR spectroscopy

**Fig 6:**
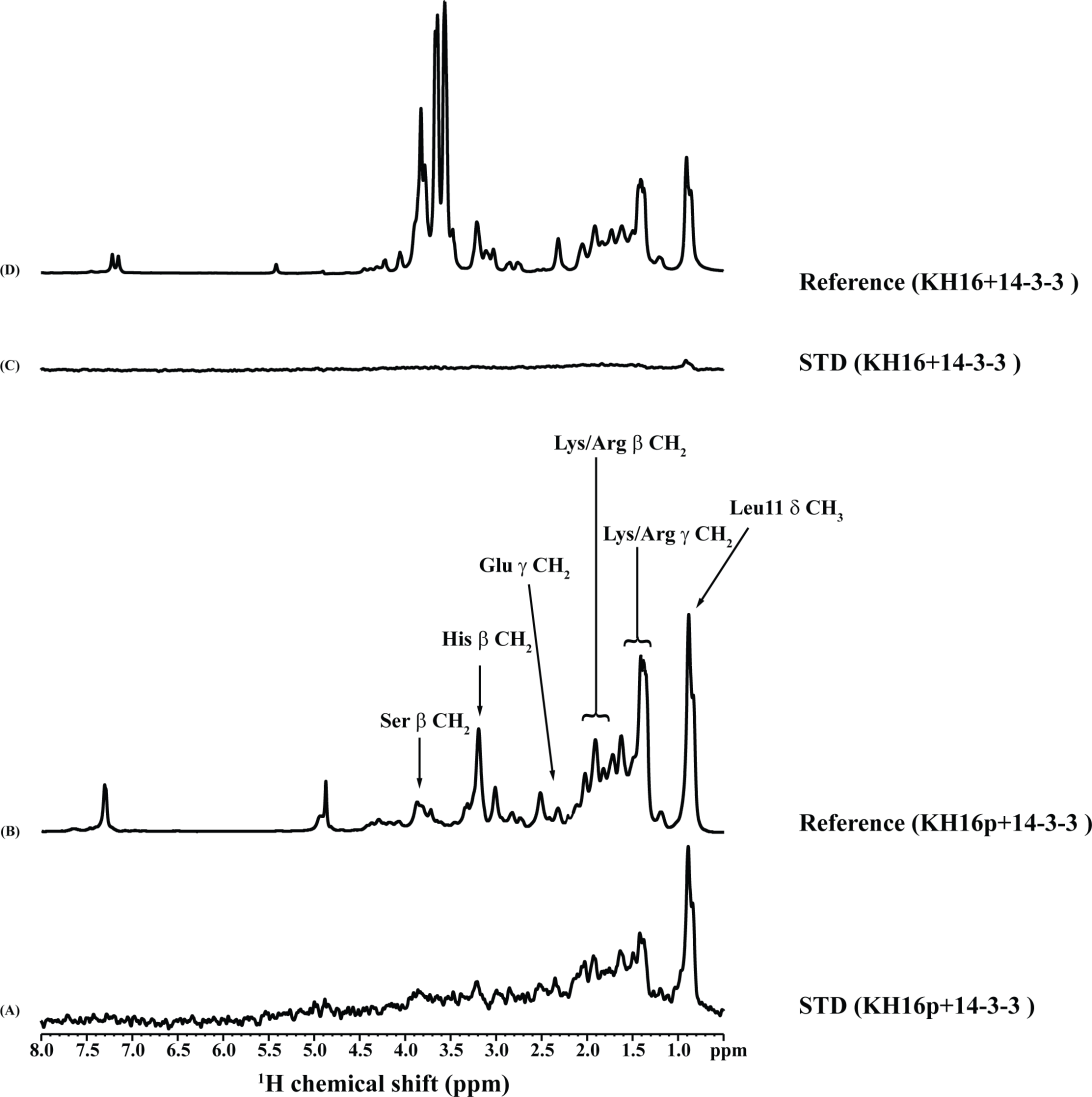
STD NMR study of KH16p and KH16 with 14-3-3ζ. **(A)** One-dimensional ^1^H NMR reference spectrum of KH16p in the presence of 14-3-3ζ at 500:1 molar ratio. **(B)** The absence of STD signals in the aromatic region and the prominent STD signals in the aliphatic region demonstrate the close proximity of few residues of KH16p to the 14-3-3ζ protein. **(C)** One-dimensional ^1^H NMR reference spectrum of KH16 in the presence of 14-3-3ζ at 500:1 molar ratio. **(D)** STD spectrum of KH16. The STD NMR experiments were carried out in aqueous solution on a Bruker AVANCE III 500 MHz spectrometer at 288 K.

STD NMR is a widely used technique to identify the important groups or residues of a ligand molecule that are in close proximity to the macromolecule [41]. It should be noted that the interaction between KH16p and 14-3-3ζ satisfies the conditions for STD NMR experiments since the KH16p undergoes fast to intermediate exchange between free and bound state, as evident from the micro-molar K_D_ value measured by ITC. Therefore, to understand the localization of KH16p with 14-3-3ζ protein as well as residue specific binding epitope, STD NMR has been used here. Fig 6A and 6B show the one-dimensional reference and STD NMR spectra of KH16p in the presence of 14-3-3ζ protein, respectively. The control STD spectrum of KH16p without 14-3-3ζ protein did not show any detectable STD signals (data not shown), whereas it showed few peaks in the aliphatic region in the presence of protein (Fig 6B). This successfully establishes the selective saturation of protein signal which propagates to the bound ligand via spin diffusion [41]. Moreover, a 1:500 molar ratio of Protein:Ligand was chosen to accomplish efficient magnetization transfer from the protein to the ligand in its bound state [40]. The strongest STD effect was observed at ~0.9 ppm which corresponds to L11δ CH_3_. As can be seen, the methylene (CH_2_) protons of L, R and K exhibit moderate STD signals. A relatively lower STD effect was observed for the beta protons of S, H and E residues. This result clearly indicates the close proximity of these residues in the vicinity of 14-3-3ζ protein. On the other hand, aromatic ring protons of H did not show any STD signals in the presence of 14-3-3ζ protein, indicating their distant position from the 14-3-3ζ protein. However, considerable resonance overlap for the aliphatic proton resonances prohibited the quantitative elucidation of the hydrophobic amino acid residues. Therefore, group epitope mapping based on ambiguous investigation of STD effects for these residues cannot be performed [56]. Collectively, our STD NMR results demonstrate that KH16p exhibits intimate interactions with the 14-3-3ζ protein, whereby L, K, R, S, H and E residues of KH16p peptide make the closer association. Importantly, STD spectra of KH16 with 14-3-3ζ protein did not show any peaks in both the aromatic and the aliphatic regions (Fig 6D). This indicates that KH16 does not bind to the 14-3-3ζ protein, as it lacks the phosphorylation at S621 residue. This distinction clearly validates our ITC-based hypothesis that dictates the importance for binding of the phosphate group at S621 residue.

### *In-silico* interaction of KH16p peptide with 14-3-3ζ protein

**Fig 7:**
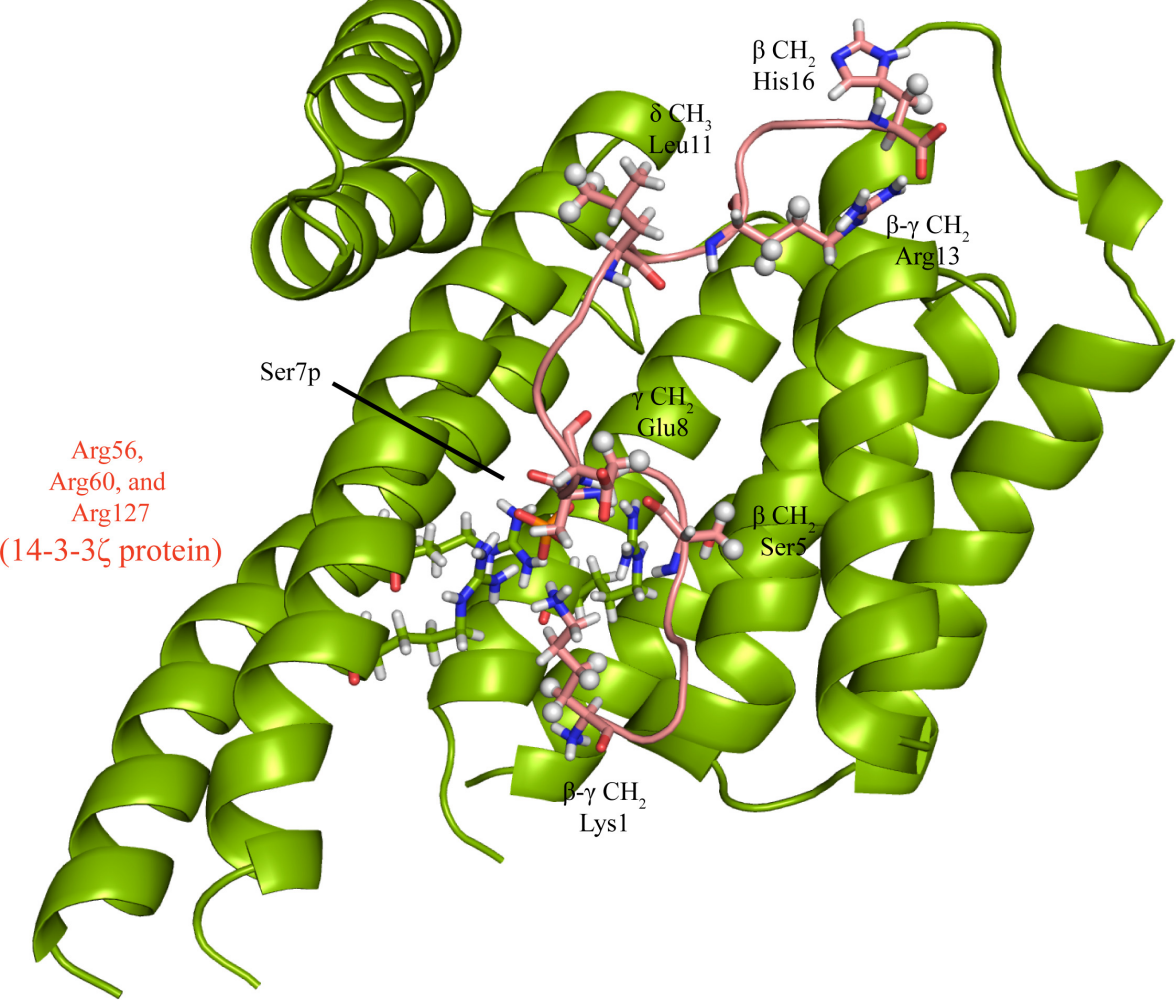
Overview of docked complex of the peptide KH16p oriented over 14-3-3ζ protein. Sphere representation of protons indicates their presence in close proximity of peptide atoms, in conjunction with data obtained from STD NMR. Relative orientation of negatively charged phosphate group (pS7) surrounded by positively charged residues like R56, R60, and R127 is shown.

The conformation of KH16p peptide was predicted to be a random coil with the help of simulated annealing procedure. It was found that an electrostatic interaction exists between the N-terminal K1 and the phosphate group of S7 (hereafter denoted as pS7), which mediates the structural stability of the peptide. Furthermore, the peptide was not found to adopt any particular secondary structure in the course of conformational sampling. Similar assumptions were also derived from CD spectroscopy, where the peptide was found to display negative minima at ~195 nm in aqueous solution. Identification of most probable peptide conformation for further interaction study with protein was performed on the basis of energy evaluation. The lowest energy state of peptide was used for docking with the 14-3-3ζ protein using Patchdock server. It should be noted that more than 20 structures were obtained as a result of the lowest energy criteria. Hence, we considered the STD NMR results as a reference for structure validation of these complexes. Negatively charged pS7 (pS621) of KH16p peptide was found to be in close proximity to the positively charged side chain of R56 and R60 of 14-3-3ζ protein. This renders a strong electrostatic interaction between peptide and protein, thereby anchoring the interaction within the complex. Apart from this, the docked complex was also found to be stabilized with CH_3_-π and π-stacking interactions between peptide residues and corresponding protein residues. The details of these interactions along with the measured distances are presented in **Table A in S1 File**. A careful inspection helps in identifying a model where the exact side chain atoms of the peptide are in close proximity to protein, in conjunction with the STD NMR data (Fig 7). Particularly, β CH_2_ of S5 (S619), γ CH_2_ of E8 (E622), δ CH_3_ as well as β CH_2_ of H12 (H626) were found within 4 Å of 14-3-3ζ protein atoms. Similarly, β/γ CH_2_ of K1 (K615) and β/γ CH_2_ of R13 (R627) were found in the vicinity of protein atoms as revealed by STD NMR. Interestingly, the peptide orientation is relatively similar to that reported recently, where R56 of 14-3-3ζ was found to be involved in an electrostatic interaction with the phosphate group of pS621 of C-Raf (pS7 of KH16p) [57].

### Behavior of peptide with protein in explicit solvent conditions

MD simulation helps in identifying the dynamics behavior of KH16p and 14-3-3ζ protein in solvent conditions. Although a strong interaction was found throughout the course of simulation time period, no change in the secondary structure of 14-3-3ζ protein was observed. Likewise, no evidence of any structural adaptation towards a particular secondary structure for the peptide was observed in the course of interaction with the protein. It was also evidenced that the interaction between the negatively charged phosphate group and that of positively charge R56 and R60 is maintained throughout the course of MD simulation. The N-terminus portion of the peptide was thus found to be in the binding pocket of 14-3-3ζ, which is mainly governed by the mentioned electrostatic interaction. On the contrary, the C-terminus portion of the peptide is found to be more exposed towards the solvent, as evidenced after 7 ns time frame. Mainly, the stretch from R13 (R627) to H16 (H630) was found to be aligned towards the solvent as evidenced from the trajectory, which tends to drive the C-terminus portion away from protein. In addition, the side chain orientation of Trp residues (of 14-3-3ζ protein) was also monitored, which infers crucial information, particularly that the protein contains two tryptophan residues (W59 and W229) in the globular structure. Interestingly, W229 is present in close proximity to the peptide (**Table A in S1 File**). In the course of MD simulation, the side chain orientation of W229 was found to be perturbed due to the impact of peptide interaction. On the other hand, the orientation of W59 remained undisturbed. The relative positional changes for the tryptophan side chains are shown in **Fig D in S1 File**. Interestingly, these results are consistent with those of the fluorescence spectra, where a blue shift of 5 nm was observed upon titration with peptide (**Fig D in S1 File**). Also, the slight decrease in intensity can be rationalized in terms of few electrostatic interactions of the polar side chains of 14-3-3ζ protein with the phosphate group of the KH16p peptide (**Fig E in S1 File**). These results indicate that the perturbation in side chain orientation of W229 is mainly due to interaction with I2 (I616) of KH16p peptide.

**Fig 8:**
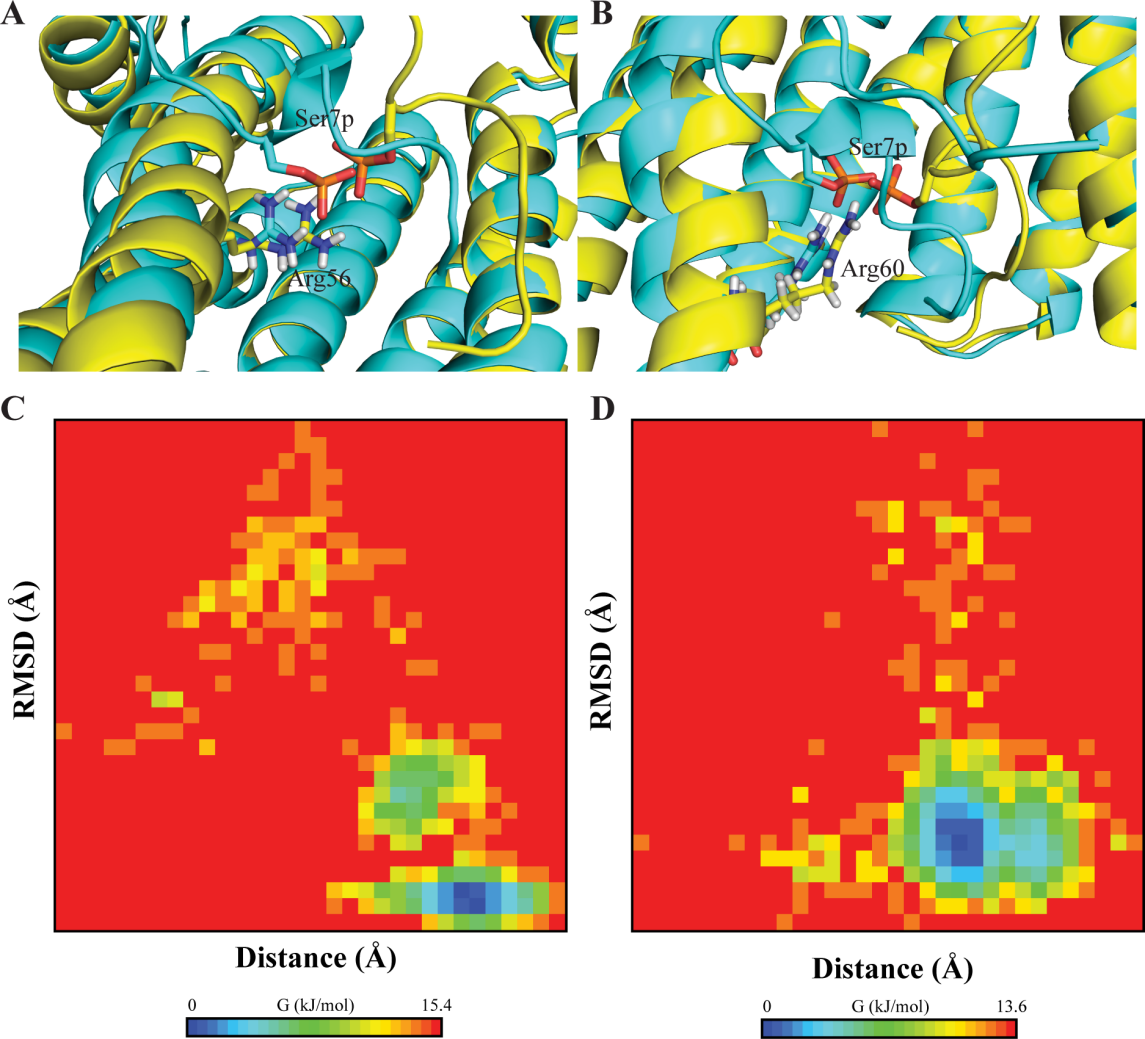
Energy landscape (bi-plot) of the interaction between negatively charged phosphate group (pS7) and positively charged residues R56 (A, C) and R60 (B, D). Collective variables were considered as the root-mean squared deviation (y-axis) and the distance between the interacting atoms (x-axis).

Our MD simulation data fit well with the STD NMR data where the side chain of S5 (S619), E8 (E622) and H16 (H630) are found to interact with nearby residues of protein. H16 (H630), which was found to be involved in a weak interaction with protein atoms, is relatively flexible as compared to the docked initial point. The same is also conferred in STD NMR, where apparently a weak signal is found for transfer of magnetization. Additionally, the side chain atoms of positively charged residues such as K1 (K615), R4 (R618) and R13 (R627) were found to be in close proximity to protein atoms, yet no stable electrostatic interactions were found to be conserved in the course of simulation. Overall, the dynamic behavior of peptide-protein complex highlights the crucial importance of phosphate group in mediating the contact. Energy landscape analysis was performed with respect to the atomic information obtained through MD simulations in conjunction with the coordinates (Fig 8). A bi-plot representation was presented considering the projection of RMSD and distance between negatively charged phosphate group and positively charged side chain of Arg residues (R56 and R60). The preparation measures for such analysis were similar to previously published report [58]. Both parameters (RMSD and distance) were taken as collective variable on the Y and X axes respectively. The local minimum obtained suggests that the energy components of the complex are stable owing to such electrostatic interaction. Further biochemical implications with respect to regulation of 14-3-3ζ protein functioning with the peptide can be derived on the basis of such strong interaction.

## Discussion

The RAF kinase performs essential tasks in several cellular processes including cell cycle progression, proliferation, differentiation and apoptosis [59] in which phosphorylation events play a crucial role. The major phosphorylation sites of C-Raf are S259 and S621, which are located at the corresponding regulatory domain and C-terminus of C-Raf’s kinase domain, respectively. Phosphorylation at these sites is a prerequisite for C-Raf interactions with the 14-3-3 family of proteins. 14-3-3 proteins have widespread and versatile biological functions, some of which are cell-cycle regulation, protein trafficking, cytoskeletal dynamics, transcription, stress responses and apoptosis [60]. From a structural viewpoint, the 14-3-3 family of proteins constitutes dimeric helical units where each of the monomers provides an amphipathic groove that accommodates the mostly phosphorylated interaction motifs of their partner protein such as C-Raf [61,62]. All isomers of 14-3-3 protein family primarily recognize three high-affinity phosphorylation-dependent binding motifs: (1) RSX**pS/T**XP (mode 1), (2) RXF/YX**pS/T**XP (mode 2), and (3) carboxyl terminal pS/TXCOOH (mode 3), where **pS/T** represents a phospho serine/threonine and X is any non-proline amino acid [61,63–65]. However, few non-phosphorylated motifs with deviation from above canonical sequences have also been reported [66]. Divergent binding modes were postulated from different computational and experimental approaches, which still need to be further elucidated [66–68]. To date, three 14-3-3ζ binding sites in C-Raf have been revealed as phosphorylated: S233, S259, and S621. 14-3-3ζ binding to S233 and S259 residues was found to be inhibitory, whereas binding to S621 residue activates C-Raf [13,15]. This has also been proved by mutational analysis that shows the exchange of S259 by alanine or aspartic acid residues resulted in enhancement of kinase activity. In contrast, mutation of S621 to alanine resulted in a protein that could no longer be activated by growth factor stimulation [15,22,48,69–72]. A high affinity (K_D_ = 16.7 μM) binding was found for pS259 peptide with 14-3-3ζ protein along with a low-affinity binding (K_D_ = 171 μM) with pS233, thus suggesting association of a dimeric 14-3-3ζ protein at these residues (K_D_ = 0.346 μM) [17]. The 50-fold lower K_D_ obtained for the di-phosphorylated peptide compared to that of high-affinity peptide suggests a positive cooperation between S233 and S259, which is further established by X-ray crystallography studies [17]. On the other hand, biochemical studies have shown an interaction of S621 C-Raf protein with 14-3-3ζ [73–76], and the importance of its nearby (±3) residues was elucidated with a phosphorylated peptide-based approach. In that study, the *K*_*i*_ for interaction between RSApSEP peptide and 14-3-3η was found to be 1270 nM [61]. Here, using a larger motif of 16 amino acids (KH16p from C-terminus of C-Raf 615–630 phosphorylated at S621 residue), we have found, based on the ITC experiment and ^31^P NMR spectroscopy, a high-affinity interaction between 14-3-3ζ and KH16p peptide with a *K*_*D*_ value of 17.33 μM, which is similar to the previously reported result for a motif designed around S259 [17]. The bound peptide does not adopt any particular secondary structure; rather, it adopts an extended conformation with a turn at the C-terminus, owing to the presence of proline residue. This sequence usually falls under mode 1 binding sequence motif RSX**pS/T**XP. Initially, circular dichroism experiments confirm the binding with 14-3-3ζ protein with retention of helicity for the protein unit. We have also performed thermal denaturation study of the protein alone and with the addition of peptide. Interestingly, we have observed increased melting temperature of 14-3-3ζ protein in the presence of KH16p peptide. These results indicate that the presence of KH16p peptide adds to the structural stability of the 14-3-3ζ protein against thermal denaturation.

The melting data is in good agreement with the literature values, where different isoforms (γ, β, ε, τ, σ) of 14-3-3 protein were subjected to thermal denaturation and were found to have a melting temperature range between 330 K to 337 K [77]. However, the peptide unit did not attain any significant structural transition upon protein binding, which in our case was also further supported by MD simulation. The ITC-derived binding affinity is in the micromolar range with 14-3-3ζ protein, which correlates well with previously published reports on other phosphopeptide motifs [57]. Our ITC data also demonstrate that the interaction with non-phosphorylated peptide (KH16) is relatively weak and the corresponding K_D_ value is in the millimolar range. The differences between the interactions of KH16p and KH16 peptides were also further elucidated by STD NMR that identified crucial residues of phosphopeptide motifs present in close proximity to 14-3-3ζ protein. These results were further confirmed by molecular modeling analysis, where despite the presence of strong interaction, the peptide was not found to attain any specific secondary structure. The interaction between protein and peptide was mainly governed by the electrostatic interaction between R56 and R60 of protein and phosphate group of the pS621 peptide. Other interacting partners were also found to mediate polar, π- π, and CH_3_-π type interactions. In addition to R56 and R60, other polar interactions were observed between protein atoms with corresponding nearby peptide residues. Briefly, carbonyl group of N224 (of 14-3-3ζ) makes a polar contact with amino group of N617 (N3 of KH16p), and carbonyl group of N224 makes a polar contact with hydroxyl group of S619 (S5 of KH16p). The amino group of K49 in protein was also found to be involved in a polar interaction with phosphate moiety of (S621) S7 of the peptide. The π-π interaction between phenyl ring of Y211 (of 14-3-3ζ) and imidazole ring of H630 (H16 of KH16p) plays an important role in stabilizing the complex. Likewise, a CH_3_-π type interaction was found to exist between phenyl ring of W228 in protein and methyl group of (I616) I2 in the peptide. A minute perturbation in side chain orientation of Trp residue is also revealed from the theoretical study, which is supported by a minute blue shift (5 nm) in fluorescence spectra. 14-3-3ζ protein can be considered as a W- shaped dimeric unit, where each subunit contains nine antiparallel α-helices. The phosphopeptide binding domain of 14-3-3ζ protein is constituted of several basic residues, such as K49, R56 and R127 along with some contribution from Y128 side-chain hydroxyl group [78]. Here, KH16p also adopts a similar conformation and binding interaction with its protein partner, as already discussed in the Results section.

Phosphorylation-dependent 14-3-3ζ binding to C-RAF's S621 accounts for its increased kinase activity. The 16-residue peptide investigated here is shown to have a strong binding affinity to the 14-3-3ζ protein. From a biophysical viewpoint, we have obtained crucial information regarding the 14-3-3ζ protein and the peptide itself, such as a high affinity binding from ITC and ^31^P NMR experiments, important residues of KH16p in close proximity to 14-3-3ζ from the STD NMR experiments as revealed by docking studies, structural modification in 14-3-3ζ W228 from MD simulation supported by fluorescence experiments, and structural perturbation in KH16p peptide from the CD experiments. Collectively, our study shows that the thermodynamically stable interaction between C-terminus region of C-Raf and 14-3-3ζ protein might ensure a crucial protection mechanism for the phosphate residue at S621 of C-Raf kinase. 14-3-3ζ binding to this residue might position this flexible region in kinase active conformation. Our biophysical data could now explain previously observed weak interaction between 14-3-3ζ and S621 phosphorylation-deficient C-Raf mutants, but further elucidation is required to explain the exact role of such strong interaction between 14-3-3ζ and C-Raf kinase in MAPK pathway.

## Supporting Information

S1 File Table A in S1 file. **Details of interacting partner from KH16p peptide and 14-3-3ζ protein conferred from docked structures**. The details are categorized as polar, CH_3_-π, and π-stacking interactions along with measured distance values. **Figure A in S1 file. Relative % of secondary structures of 14-3-3ζ protein and KH16p peptide alone and in complex, computed from CD spectra recorded at 298 K by CDNN software**. (**A)** Relative secondary structure of 14-3-3ζ protein alone (black) and in the presence of KH16p peptide (red). (**B)** Relative secondary structure of KH16p peptide alone (black) and in the presence (red) of 14-3-3ζ protein. **Figure B in S1 file. CD melting study of 14-3-3ζ protein with and without KH16p.** Thermal melting study of 14-3-3ζ protein in the absence and presence of KH16p peptide (1:2 molar ratio) with CD spectrophotometer. All experiments were performed in 10 mM Na_2_HPO_4_ with 100 mM NaCl (pH 6.1) buffer at 298K. **Figure C in S1 file. Sequential assignment for KH16p in presence of 14-3-3ζ protein.** Fingerprint region of NOESY spectra showing the NOE cross peaks between CαH-NH resonances (red) overlaid on TOCSY spectrum (blue). **Figure D in S1 file. Relative change in position of Trp59 and Trp228 of 14-3-3ζ protein upon interaction with KH16p peptide correlated with fluorescence spectra**. **(A)** The comparison of the side chain orientation was made with reference to structural changes visualized from MD simulation. Color code for 14-3-3ζ protein is green (0 ns) and pink (15 ns). Comparison of structural change is correlated with Fluorescence spectrum. **(B)** Fluorescence emission spectra for the 14-3-3ζ protein alone and in presence of KH16p protein in a 1:5 molar ratio demonstrate ~5 nm blue shift. **Figure E in S1 file. Pymol session containing the starting and final model (pdb format) snapshot from molecular dynamics simulation**. Phosphorylated residue (Ser7p) interacting with Arg56 and Arg60 are highlighted in sticks representation.
(PDF)
