# Supplementary material for: Biophysical Characterization of Essential Phosphorylation at the Flexible C-Terminal Region of C-Raf with 14-3-3ζ Protein
Source: PLoS One. 2015 Aug 21;10(8):e0135976. doi: 10.1371/journal.pone.0135976 (PMC4546627; doi:10.1371/journal.pone.0135976)
Supplement: S1 File — Details of interacting partner from KH16p peptide and 14-3-3ζ protein conferred from docked structures. The details are categorized as polar, CH3-π, and π-stacking interactions along with measured distance values. Figure A in S1 file. Relative % of secondary structures of 14-3-3ζ protein and KH16p peptide alone and in complex, computed from CD spectra recorded at 298 K by CDNN software. (A) Relative secondary structure of 14-3-3ζ protein alone (black) and in the presence of KH16p peptide (red). (B) Relative secondary structure of KH16p peptide alone (black) and in the presence (red) of 14-3-3ζ protein. Figure B in S1 file. CD melting study of 14-3-3ζ protein with and without KH16p. Thermal melting study of 14-3-3ζ protein in the absence and presence of KH16p peptide (1:2 molar ratio) with CD spectrophotometer. All experiments were performed in 10 mM Na2HPO4 with 100 mM NaCl (pH 6.1) buffer at 298K. Figure C in S1 file. Sequential assignment for KH16p in presence of 14-3-3ζ protein. Fingerprint region of NOESY spectra showing the NOE cross peaks between CαH-NH resonances (red) overlaid on TOCSY spectrum (blue). Figure D in S1 file. Relative change in position of Trp59 and Trp228 of 14-3-3ζ protein upon interaction with KH16p peptide correlated with fluorescence spectra. (A) The comparison of the side chain orientation was made with reference to structural changes visualized from MD simulation. Color code for 14-3-3ζ protein is green (0 ns) and pink (15 ns). Comparison of structural change is correlated with Fluorescence spectrum. (B) Fluorescence emission spectra for the 14-3-3ζ protein alone and in presence of KH16p protein in a 1:5 molar ratio demonstrate ~5 nm blue shift. Figure E in S1 file. Pymol session containing the starting and final model (pdb format) snapshot from molecular dynamics simulation. Phosphorylated residue (Ser7p) interacting with Arg56 and Arg60 are highlighted in sticks representation. (PDF) [file pone.0135976.s001.pdf]

Supporting Information

Table A in S1 File. Details of interacting partner from KH16p peptide and 14-3-3ζ protein conferred from docked structures. The details are categorized as polar, CH_3-π, and π-stacking interactions along with measured distance values.

Interaction type	KH16p	14-3-4 ζ protein	Distance (Å)
**Polar Interaction**	Phosphate moiety Ser7	Guanidino group Arg60	1.9
	Phosphate moiety Ser7	Guanidino group Arg56	3.9
	Amino group of side chain Asn3	Carbonyl group of side chain Asn224	2.5
	Phosphate moiety Ser7	Amino group of Lys49	4.2
	Hydroxyl group of Ser5	Carbonyl group of Asn224	2.6
**CH_3-π Interaction**	Methyl group of side chain Ile2	Phenyl ring of side chain Trp228	3.1
**π-stacking interaction**	Imidazole ring of side chain His16	Phenyl ring of side chain Tyr211	3.8

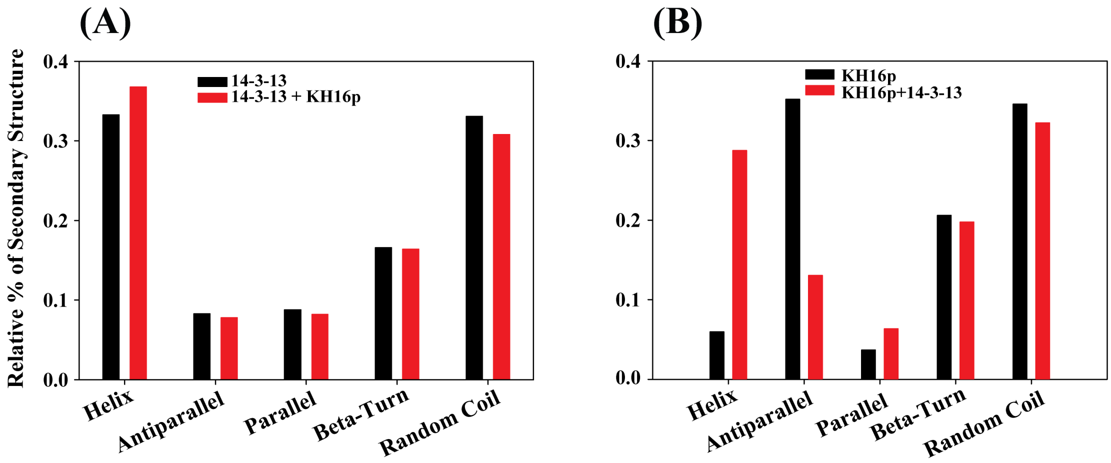

Fig A in S1 file. Relative % of secondary structures of 14-3-3ζ protein and KH16p peptide alone and in complex, computed from CD spectra recorded at 298 K by CDNN software. (**A)** Relative secondary structure of 14-3-3ζ protein alone (black) and in presence of KH16p peptide (red). (**B)** Relative secondary structure of KH16p peptide alone (black) and in presence (red) of 14-3-3ζ protein.

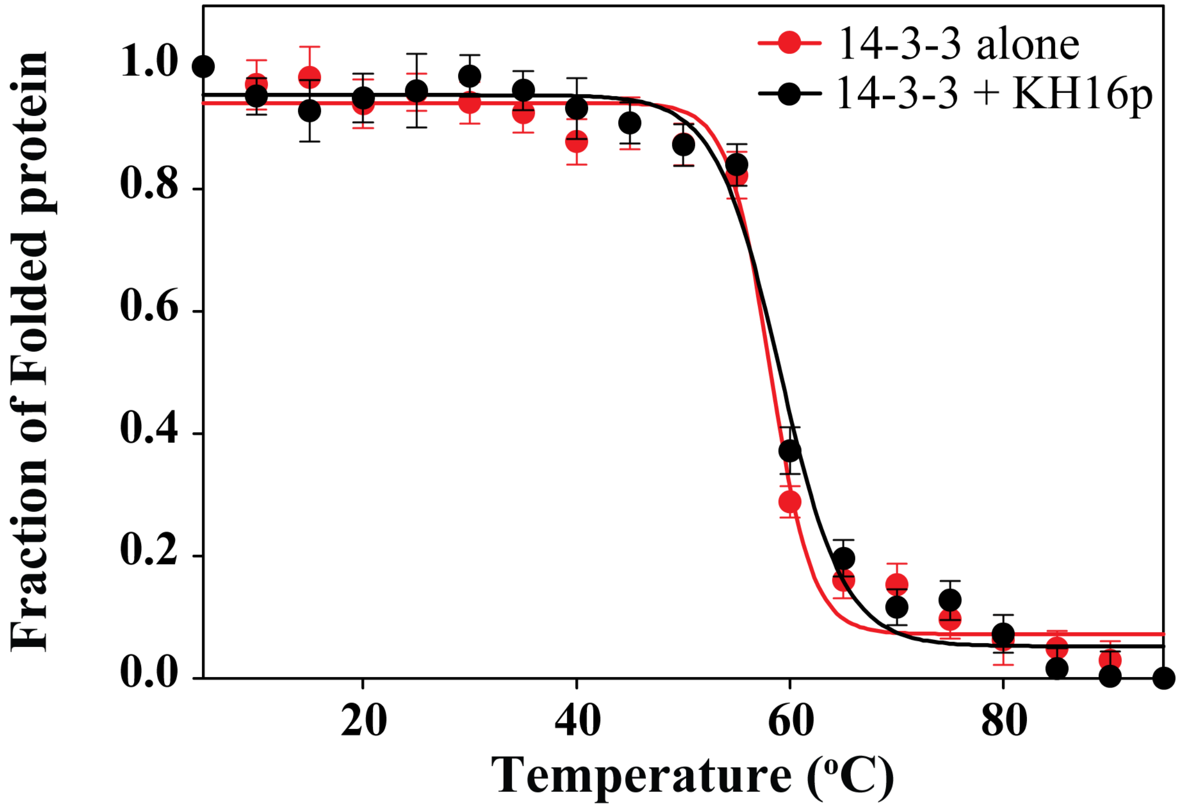

Fig B in S1 file. CD melting study of 14-3-3ζ protein with and without KH16p. Thermal melting study of 14-3-3ζ protein in presence and absence of KH16p peptide (1:2 molar ratio) with CD spectrophotometer. All experiments were perfomred in 10 mM Na_2HPO_4 with 100 mM NaCl (pH 6.1) buffer at 298 K.

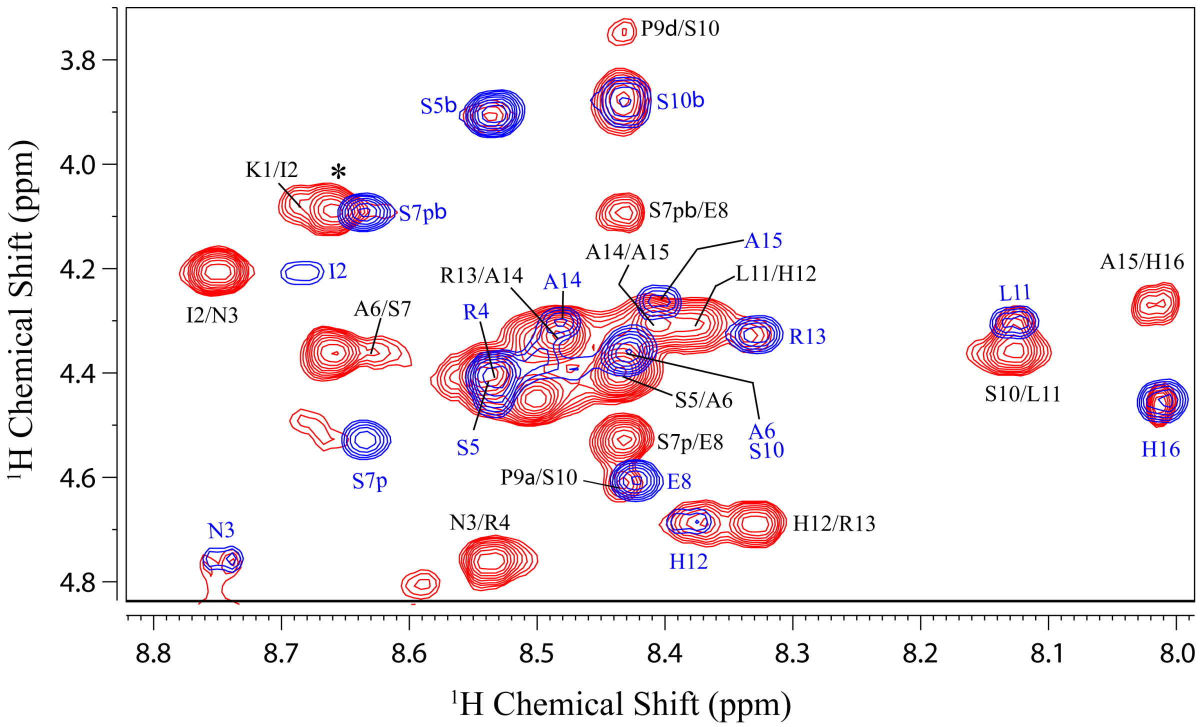

Fig C in S1 file. Sequential assignment for KH16p in presence of 14-3-3ζ protein. Fingerprint region of NOESY spectra showing the NOE cross peaks between CαH-NH resonances (red) overlaid on TOCSY spectrum (blue).

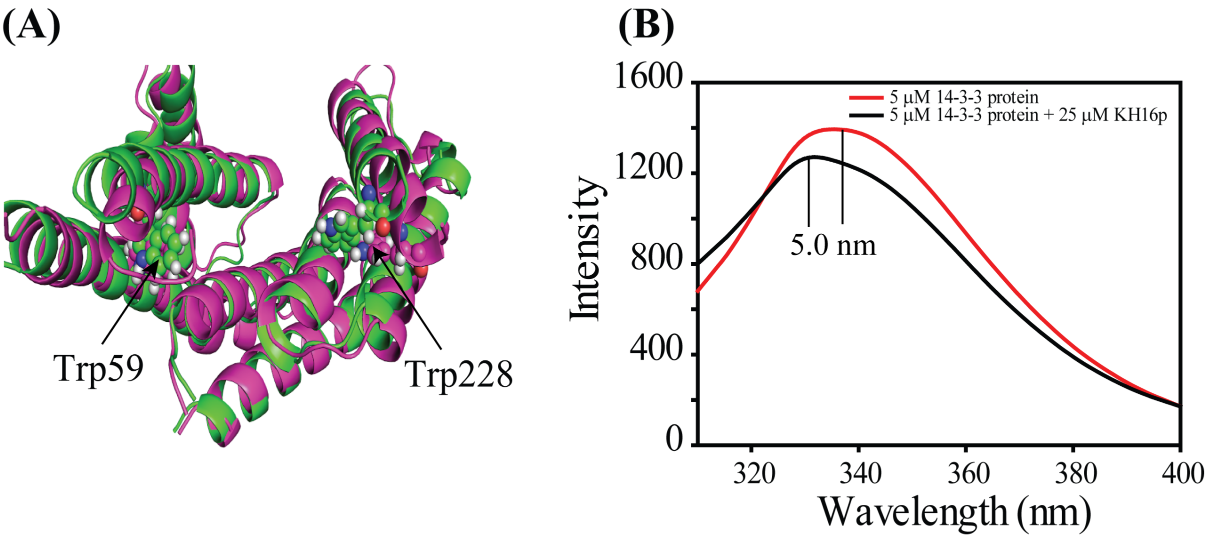

Fig D in S1 file. Relative change in position of Trp59 and Trp228 of 14-3-3ζ protein upon interaction with KH16p peptide correlated with fluorescence spectra. **(A)** The comparison of the side chain orientation was made with reference to structural changes visualised from MD simulation. Color code for 14-3-3ζ protein is green (0 ns) and pink (15 ns). Comparison of structural change is correlated with Fluorescence spectrum. **(B)** Fluorescence emission spectra for 14-3-3ζ protein alone and in presence of KH16p protein in a 1:5 molar ratio demonstrating ~5 nm blue shift.
